# Regulation of Nucleolar Dominance in *Drosophila melanogaster*

**DOI:** 10.1534/genetics.119.302471

**Published:** 2020-03-03

**Authors:** Natalie Warsinger-Pepe, Duojia Li, Yukiko M. Yamashita

**Affiliations:** * Department of Molecular and Integrative Physiology, University of Michigan, Ann Arbor, Michigan; † Life Sciences Institute, University of Michigan, Ann Arbor, Michigan; ‡ Molecular, Cellular and Developmental Biology, University of Michigan, Ann Arbor, Michigan; § Cell and Developmental Biology, University of Michigan, Ann Arbor, Michigan; ** Howard Hughes Medical Institute, University of Michigan, Ann Arbor, Michigan 48109

**Keywords:** nucleolar dominance, rDNA, *Drosophila*

## Abstract

In eukaryotic genomes, ribosomal RNA (rRNA) genes exist as tandemly repeated clusters, forming ribosomal DNA (rDNA) loci. Each rDNA locus typically contains hundreds of rRNA genes to meet the high demand of ribosome biogenesis. Nucleolar dominance is a phenomenon whereby individual rDNA loci are entirely silenced or transcribed, and is believed to be a mechanism to control rRNA dosage. Nucleolar dominance was originally noted to occur in interspecies hybrids, and has been shown to occur within a species (*i.e*., nonhybrid context). However, studying nucleolar dominance within a species has been challenging due to the highly homogenous sequence across rDNA loci. By utilizing single nucleotide polymorphisms between *X* rDNA and *Y* rDNA loci in males, as well as sequence variations between two *X* rDNA loci in females, we conducted a thorough characterization of nucleolar dominance throughout development of *Drosophila melanogaster*. We demonstrate that nucleolar dominance is a developmentally regulated program that occurs in nonhybrid, wild-type *D. melanogaster*, where *Y* rDNA dominance is established during male embryogenesis, whereas females normally do not exhibit dominance between two *X* rDNA loci. By utilizing various chromosomal complements (*e.g.*, *X/Y*, *X/X*, *X/X/Y*) and a chromosome rearrangement, we show that the short arm of the *Y* chromosome including the *Y* rDNA likely contains information that instructs the state of nucleolar dominance. Our study begins to reveal the mechanisms underlying the selection of rDNA loci for activation/silencing in nucleolar dominance in the context of nonhybrid *D. melanogaster*.

RIBOSOMAL DNA (rDNA), genes encoding the catalytic RNA components of ribosomes, is highly repetitive (100s–1000s of copies) and often exists as multiple loci on separate chromosomes (*e.g*., two loci in *Drosophila melanogaster*, four in *Arabidopsis*, 10–12 in *Mus musculus*, ∼10 in *Homo sapiens* per diploid genome) (Long and Dawid 1980; Pontes *et al.* 2004). This expansive copy number may come as no surprise, considering that the transcription of rDNA accounts for ∼60% of the total transcription of a metabolically active cell (Moss and Stefanovsky 2002). The regulation of ribosomal RNA (rRNA) expression is critically important for adjusting cellular energetic expenditure: when nutrients are low, rRNA synthesis is downregulated, whereas the opposite occurs when nutrients are high or growth rate is increased (*e.g.*, in cancer) (Smetana and Busch 1964; Busch *et al.* 1979; Ghoshal *et al.* 2004; Grewal *et al.* 2005; Murayama *et al.* 2008; Aldrich and Maggert 2015). Accordingly, transcription of rRNA is expected to require precise regulation.

A phenomenon called nucleolar dominance, whereby individual rDNA loci are either entirely expressed or silenced, is proposed to be a mechanism that regulates the dosage of rRNA (Preuss and Pikaard 2007). Nucleolar dominance has been noted to be one of the largest epigenetic mechanisms, second only to *X* inactivation in eutherian mammals (Pikaard 2000). Nucleolar dominance was originally discovered in interspecies hybrids [*i.e.*, *Xenopus* hybrids (Cassidy and Blackler 1974), *Arabidopsis* hybrids (Chen *et al.* 1998), *Drosophila* hybrids (Durica and Krider 1977; Goodrich-Young and Krider 1989; Oliveira *et al.* 2006), and mouse-human hybrid cell lines (Croce *et al.* 1977)], where rDNA loci inherited from one species are preferentially expressed and those from the other are silenced. Later, nucleolar dominance was shown to occur within a species (*i.e.*, nonhybrid context) (Lewis *et al.* 2004; Greil and Ahmad 2012; Zhou *et al.* 2012), indicating that nucleolar dominance is a mechanism to regulate rRNA expression/dosage instead of a result of interspecies incompatibility.

Nucleolar dominance has been thoroughly studied in *Arabidopsis*, both in *A. suecica* (the interspecies hybrid between *A. thaliana* and *A. arenosa*) as well as nonhybrid *A. thaliana* (Pontes *et al.* 2007; Earley *et al.* 2010). In both cases, nucleolar dominance is gradually established during development, where seedling cotyledons express rRNA from all rDNA loci (*i.e.*, “codominance”), transitioning to preferential expression of certain loci in mature tissues (Pontes *et al.* 2007; Earley *et al.* 2010). Several mechanisms have been shown to mediate the silencing of chosen rDNA loci, including small interfering RNAs (siRNAs) (Pontes *et al.* 2006; Preuss *et al.* 2008), DNA methylation (Chen *et al.* 1998; Lawrence *et al.* 2004; Pontes *et al.* 2006; Preuss *et al.* 2008; Costa-Nunes *et al.* 2010; Earley *et al.* 2010), histone methylation (Earley *et al.* 2010; Pontvianne *et al.* 2012), and histone deacetylation (Probst *et al.* 2004; Earley *et al.* 2006; Earley *et al.* 2010). These mechanisms reveal how the large-scale silencing of rDNA is implemented to achieve nucleolar dominance; however, what factor(s) influence the choice of which rDNA loci are silenced or activated remains elusive.

Nucleolar dominance is likely a widespread phenomenon across many species. For example, only a subset of rDNA loci are transcribed in human cell lines (Roussel *et al.* 1996) and human lymphocytes (Roussel *et al.* 1996; Héliot *et al.* 2000), implying that these cells also may undergo nucleolar dominance, although which loci are silenced and/or expressed remains unknown. Nucleolar dominance was found to occur in “pure species” or “nonhybrid” *D. melanogaster* larval neuroblasts, where the rDNA on the *Y* chromosome (“*Y* rDNA”) dominates over rDNA on the *X* chromosome (“*X* rDNA”), based on transcription-dependent deposition of GFP-tagged histone H3.3 onto the active rDNA locus (*i.e.*, the *Y* rDNA locus) (Greil and Ahmad 2012) and the presence of a secondary constriction of the active rDNA locus observed on the condensed mitotic chromosomes (Zhou *et al.* 2012). These methods relied on readily available mitotic chromosomes, leaving the assessment of nucleolar dominance in other cell types (*e.g.*, those not frequently undergoing mitosis) elusive. Recently, we adapted a single nucleotide polymorphism RNA fluorescent *in situ* hybridization (SNP *in situ*) protocol and showed that nucleolar dominance (*Y* rDNA dominance) also occurs in male germline stem cells (GSCs) (Levesque *et al.* 2013; Lu *et al.* 2018). This method utilizes SNPs between the *X* rDNA and the *Y* rDNA to differentially label their products (*X*- *vs.* *Y*-derived rRNA), allowing assessment of nucleolar dominance without requiring mitotic chromosomes.

In this study, we utilized SNP *in situ* to comprehensively examine the state of nucleolar dominance in *D. melanogaster* (*i.e.*, “nonhybrid” context) during development and across different tissues. We show that nucleolar dominance in *D. melanogaster* is gradually established during development, similar to the observations in *A. thaliana*, supporting the notion that nucleolar dominance is a regulatory mechanism that occurs in nonhybrid organisms. We have further examined the state of nucleolar dominance between two *X* rDNA loci in females by isolating *X* rDNA with distinct sequences that enables RNA *in situ* hybridization to distinguish transcripts from two *X* rDNA loci. Our results show that the two *X* rDNA loci in females exhibit codominance in essentially all tissues, expanding the previous finding of codominance in female larval neuroblasts (Greil and Ahmad 2012). Moreover, by utilizing various karyotypes (*e.g.*, *X/X* females, *X/Y* males, *vs.* *X/X/Y* females) and a chromosome rearrangement strain, we show that *Y* chromosome element(s) (within *Y* rDNA as well as non-rDNA element(s) of the *Y* chromosome) may aid in the “choice” mechanism that underlies nucleolar dominance, *i.e.*, the preferential expression of the *Y* rDNA locus and the preferential silencing of the *X* rDNA locus. These results provide insights into how specific rDNA loci may be preferentially transcribed/silenced, and will provide the foundation for future studies aimed at understanding the regulation and biological meaning of nucleolar dominance.

## Materials and Methods

### Fly husbandry and strains

Unless otherwise stated, all fly stocks (see Reagent Table) were raised on standard Bloomington medium at room temperature (RT). Unless otherwise stated, all flies used for wild-type experiments were the standard laboratory wild-type strain *y^1^w^1^*, referred to as *yw*, that contains the *X* and *Y* chromosomes with mapped rDNA SNPs (Lu *et al.* 2018) (see Reagent Table). Stocks used to study female nucleolar dominance were obtained from the University of California, San Diego *Drosophila* Stock Center and the culture was established by using single-pair parents to minimize heterogeneity of rDNA within each stock.

The *X* and *Y* chromosomes from wild type (*yw*) were introduced into genotypes of interest analyzed in this study to keep the rDNA loci consistent across experiments. When it was not feasible to introduce the wild-type (*yw*) *X* and/or *Y* chromosomes into a genetic background of interest, their rDNA was sequenced to find SNPs between the *X* and *Y* rDNA and the appropriate SNP *in situ* probes were used (see Reagent Table).

### RNA *in situ* hybridization

Third instar larval or adult tissues were dissected in RNase-free 1 × PBS (phosphate buffered saline), fixed in RNase-free 4% formaldehyde, and incubated overnight in 70% EtOH at 4° to permeabilize the tissues. Embryos were collected according to a modified protocol from Wilk *et al.* (2010), by allowing parents to lay eggs on an apple-agar plate at RT for a range of collection time (3–17 hr). Embryos were transferred to glass scintillation vials with glass Pasteur pipettes and were washed of any yeast in 1 × PBS then dechorionated in 50% bleach for 30 sec and washed again in PBS. The embryos were then devitellinized and fixed in 50:50 heptane:4% RNase-free formaldehyde during vigorous, manual shaking for 20 min, then again in 50:50 heptane:methanol twice for 30 sec, washed in methanol, and then stored in methanol at −20° for at least one night before proceeding to *in situ* hybridization.

*In situ* hybridization was performed as previously described with slight modifications (Lu *et al.* 2018). In short, *X/Y* and *X/X/Y* samples were washed with wash buffer (10% formamide in 1 × SSC and 0.1% Tween-20) for 5 min, then incubated with the hybridization mix [10% formamide, 1 × SSC, 10% Dextran sulfate (w/v)] (D8906; Sigma, St. Louis, MO), 100 nM each *in situ* fluorescent probe (*X* and *Y* rDNA SNP probes), and 300 nM each mask oligo (for SNP *in situ*) overnight in a 37° water bath. Samples were then washed twice in wash buffer for 30 min each at 37° and stored in Vectashield H-1200 (Vector Laboratories, Burlingame, CA) with DAPI. Hybridization and washes for *X/X* females were performed at 42°. Images were taken using a Leica TCS SP8 confocal microscope with 63X oil-immersion objectives and processed using Adobe Photoshop software.

See Reagent Table for fluorescent *in situ* oligonucleotide probes. Unless otherwise stated, all four *X* rDNA SNP *in situ* probes and all four *Y* rDNA SNP *in situ* probes were used for each experiment visualizing *X* and *Y* rRNA. Stocks that required the use of fewer than four of the SNP *in situ* probes are listed in the Reagent Table. SNP *in situ* oligonucleotide probes were custom ordered from Biosearch Technologies (Lu *et al.* 2018). Fluorescent *in situ* oligonucleotide probes used to study female nucleolar dominance were designed using Integrated DNA Technologies Oligo Analyzer.

### Identification of SNPs in rDNA

To sequence *X* rDNA, genomic DNA was extracted from 10–15 female flies of a genotype of interest. To sequence *Y* rDNA, male flies of the genotype of interest were crossed to C(1)DX/Y female flies, which lack *X* rDNA, and 10–15 female progeny [which have the *Y* chromosome of interest and C(1)DX] was subjected to genomic DNA extraction. PCR was performed on the extracted genomic DNA to amplify three regions of the rDNA with the following primers:

18S: (forward) 5′-GAAACGGCTACCACATCTAAGG-3′ and (reverse) 5′-GGACCTCTCGGTCTAGGAAATA-3′.
ITS1: (forward) 5′-CTTGCGTGTTACGGTTGTTTC-3′ and (reverse) 5′-ACAGCATGGACTGCGATATG-3′.
28S: (forward) 5′-AGCCCGATGAACCTGAATATC-3′ and (reverse) 5′-CATGCTCTTCTAGCCCATCTAC-3′ (Lu *et al.* 2018).

PCR products were verified by agarose gel electrophoresis and purified using a PCR Purification Kit (Qiagen, Valencia, CA). Sanger sequencing was performed on the purified PCR products using the same PCR primers (University of Michigan Biomedical Research DNA Sequencing Core Facility). Sequencing data were analyzed using the free downloadable software ApE: A plasmid Editor, by M. Wayne Davis.

### Larval brain squash and DNA fluorescent *in situ* hybridization on mitotic chromosomes

We utilized a modified DNA fluorescent *in situ* hybridization protocol described previously (Larracuente and Ferree 2015; Jagannathan *et al.* 2017). In short, third instar larvae were collected and brains were dissected in 1 × PBS. Larval brains were fixed in 45:55 acetic acid:4% formaldehyde in PBS on Superfrost Plus Microscope Slides (22-037-246; Fisherbrand). The sample was then covered with a coverslip, manually squashed, and submerged in liquid nitrogen until frozen. The coverslips were quickly removed and the slides were treated with 100% ethanol at RT for 5 min. Then, 20 µl of hybridization buffer (50% formamide, 10% dextran sulfate, 2 × SSC buffer, 0.5 μM of each probe) was added to the sample, covered with a coverslip, and the sample was heat-denatured at 95° for 5 min, followed by incubation in a humid chamber in the dark overnight at RT. Samples were washed three times for 15 min in 0.1 × SSC and then mounted in Vectashield H-1200 (Vector Laboratories) with DAPI. Probe sequences are provided in the Reagent Table.

### Immunofluorescence on mitotic chromosome spreads

A protocol described by Blum *et al.* (2017) was used to conduct immunofluorescence on mitotic chromosome spreads. Briefly, larval brains from third instar larvae were dissected and incubated in 30 μl of 0.5% sodium citrate on Superfrost Plus Microscope Slides (22-037-246; Fisherbrand) for 10–20 min. Sodium citrate was gently removed using a micropipette. Then, 25 μl of 4% formaldehyde was gently added to the slide over the sample, removed with a micropipette and replaced with another fresh 25 μl of 4% formaldehyde and fixed for 4 min. During fixation, the larval brains were dissected into smaller pieces. Any imaginal discs and/or the ventral nerve cord were removed during this process. After fixation, the sample was covered with a coverslip, squashed, and submerged in liquid nitrogen until frozen. After removal of the coverslips, slides were washed in PBS for 30–60 min and incubated overnight with primary antibodies (Chicken anti-Cid, 1:200) in 3% BSA in 1 × PBST (phosphate buffered saline with 0.1% triton X-100) at 4° in a humid chamber. The slides were washed in 1 × PBST, three times for 20 min each, then incubated with secondary antibodies (A-11039, 1:200, goat anti-chicken Alexa Fluor 488; Invitrogen, Carlsbad, CA) in 3% BSA in 1 × PBST for 45 min at RT in a humid chamber in the dark. Slides were washed in 1 × PBST, three times for 20 min, and mounted in Vectashield H-1200 (Vector Laboratories) with DAPI. Antibodies are listed in the Reagent Table.

### Quantification and statistical analysis of nucleolar dominance

Nucleolar dominance was quantified manually from images generated using a Leica TCS SP8 confocal microscope. For each embryo, larval brain, imaginal disc, larval anterior midgut, and adult anterior midgut sample, one to three representative images of each tissue were captured for scoring purposes. Imaginal discs were randomly scored without intentionally excluding any imaginal disc type, therefore all imaginal discs were included in the category of “imaginal discs” for scoring purposes. *Z*-stacks were generated with maximum projections for pregastrulation embryos, larval anterior midgut, and adult anterior midgut images for scoring. Whole tissues were scored for salivary glands and larval fat bodies. All cells were identified and scored based on nuclear DAPI staining and morphology. Note that the call of dominance *vs.* codominance was straightforward, owing to consistent signal intensity across samples based on the RNA *in situ* procedure described above. The number of cells and the number of tissues scored per genotype are listed in each corresponding figure legend and in Supplemental Material, Table S1. *P*-values were calculated using an unpaired Student’s *t*-test with Welch’s correction (assuming unequal variances), with *n* representing number of tissues scored.

### Data availability

*Drosophila* strains and reagents are listed in the Reagent Table and/or above. Raw scoring data are provided in Table S1. Numerical data that are not listed in the text are available in Table S1. Reagent Table and Table S1 can be found on figshare. Supplemental material available at figshare: https://doi.org/10.25386/genetics.11910777.

## Results

### *Y* rDNA dominance is gradually established during male development

**Figure 1:**
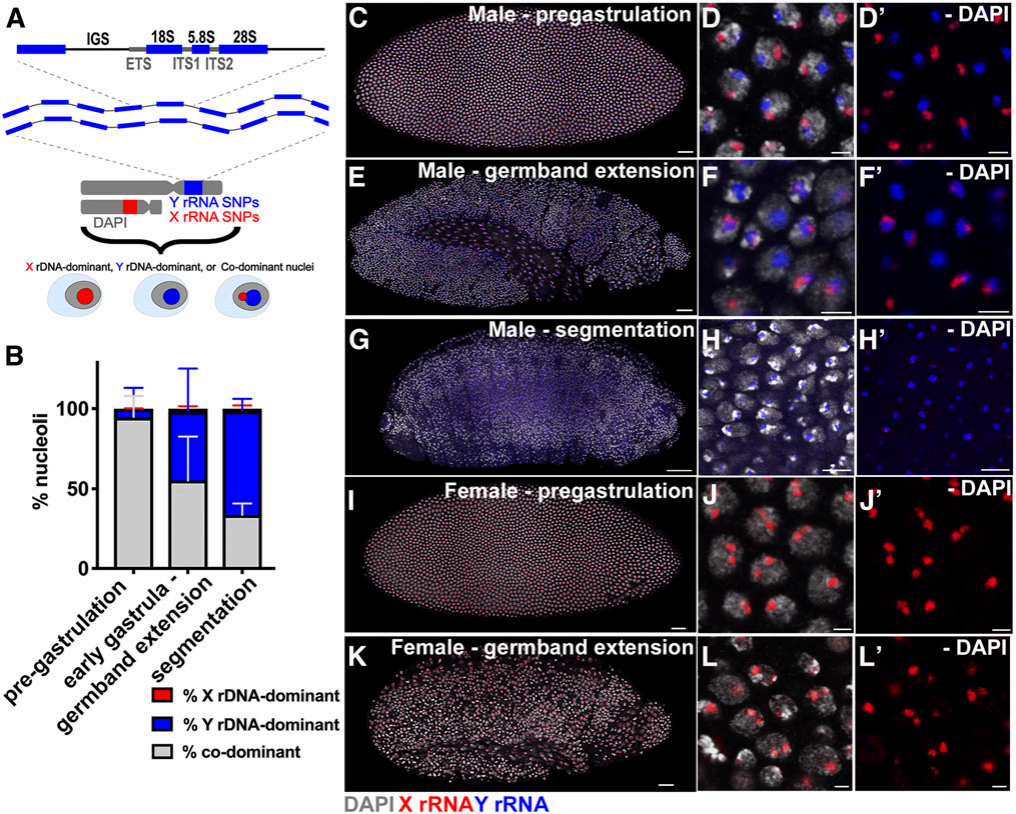
Nucleolar dominance is not established during embryogenesis in male embryos. (A) Schematic of rDNA repeats. *X* and *Y* rDNA can be distinguished by SNP *in situ* hybridization. Definition of *X* rDNA-dominant, *Y* rDNA-dominant, or codominant is shown. (B) Quantification of nucleolar dominance across embryogenesis in males: pregastrulation (*n* = 748 cells from 7 embryos), early gastrula through germband extension (*n* = 1086 cells from 12 embryos), and segmentation (*n* = 1242 cells from 10 embryos). Red = % *X* rDNA-dominant, blue = % *Y* rDNA-dominant, gray = % codominant. (C) Male pregastrulation embryo, Bar, 25 μm. (D) Zoomed image of nuclei from male pregastrulation embryo, Bar, 3 μm. (D’) No DAPI. (E) Male embryo at germband extension stage, Bar, 25 μm. (F) Zoomed image of male embryo at germband extension stage, Bar, 3 μm. (F’) No DAPI. (G) Male embryo at segmentation stage, Bar, 50 μm. (H) Zoomed image of male embryo at segmentation state, Bar, 8 μm. (H’) No DAPI. (I) Female pregastrulation embryo, Bar, 25 μm. (J) Zoomed image of female pregastrulation embryo, Bar, 3 μm. (J’) No DAPI. (K) Female embryo at germband extension stage, Bar, 25 μm. (L) Zoomed image of female embryo at germband extension stage, Bar, 3 μm. (L’) No DAPI. Red = *X* rRNA, blue = *Y* rRNA, white = DAPI. 18S, 5.8S, and 28S, rRNA coding region; ETS, external transcribed spacer; IGS, intergenic spacer; ITS, internal transcribed spacer.

Thorough characterization of nucleolar dominance within a species (*i.e.*, in the context of nonhybrids) has been limited to *A. thaliana* (Tucker *et al.* 2010). Prior analysis of nucleolar dominance in nonhybrid *D. melanogaster* has been limited to larval neuroblasts and adult male germline cells (Greil and Ahmad 2012; Zhou *et al.* 2012; Lu *et al.* 2018). To extend the analysis of nucleolar dominance in nonhybrid *D. melanogaster*, we applied the SNP *in situ* hybridization method that differentiates *X* rDNA-derived rRNA *vs.* *Y* rDNA-derived rRNA (Lu *et al.* 2018). Using this technique, we comprehensively analyzed the state of nucleolar dominance during development of *D. melanogaster* (Figure 1A). In all experiments reported in this study, *X* and *Y* chromosomes with defined rDNA SNPs from a wild-type strain (*yw*) were introduced into the genetic background of interest. Alternatively, distinct SNPs were identified by sequencing *X* and *Y* rDNA loci, if introduction of the *yw* strain sex chromosomes was complicated/impossible (see *Materials and Methods*).

We first focused on nucleolar dominance in male embryos: 48.6% of the total embryos scored (*n* = 368) contained both the *Y* rDNA and *X* rDNA SNP signals, which we deemed as male embryos. Note that not all nuclei within an embryo necessarily contained both *X* rRNA and *Y* rRNA signals, but the presence of any *Y* rRNA-containing nuclei within an embryo indicates that they are male embryos. On the contrary, 51.4% of embryos contained only *X* rDNA SNP signal in all nuclei within an entire embryo, which were deemed as female embryos. Since our SNP *in situ* probes cannot discriminate rRNA signals from two *X* rDNA loci in females, their state of nucleolar dominance cannot be determined by these experiments (Figure 1, I–L) (see below for nucleolar dominance in females). We found that in early male embryos (pregastrulating, around syncytial cycle 13–14), the majority of nuclei expressed both the *X* and *Y* rDNA (*i.e.*, codominant) (94.8 ± 13.2%) (Figure 1, B–D). It has been reported that larval neuroblasts (Greil and Ahmad 2012), male GSCs, and spermatogonia (Lu *et al.* 2018) exhibit *Y* rDNA dominance, suggesting that nucleolar dominance may be established during the course of development. To address this possibility, we examined the state of nucleolar dominance along the course of development through embryonic stages, larval development, and into adulthood. Although the pregastrulating embryos exhibited a high frequency of codominance (∼95%), we observed a decrease in the percentage of codominant nuclei, with a concomitant increase in *Y* rDNA-dominant cells as male embryos progressed through development (Figure 1, B–H). Male embryos during early gastrula or germband extension stages show 55.3 ± 27.5% codominant nuclei and 42.9 ± 27.0% *Y* rDNA dominant nuclei (Figure 1, B, E, F). Later during segmentation, codominant nuclei further decreased to 33.7 ± 7.1%, as *Y* rDNA dominant nuclei increased to 64.6 ± 7.9% (Figure 1B, G–H).

**Figure 2:**
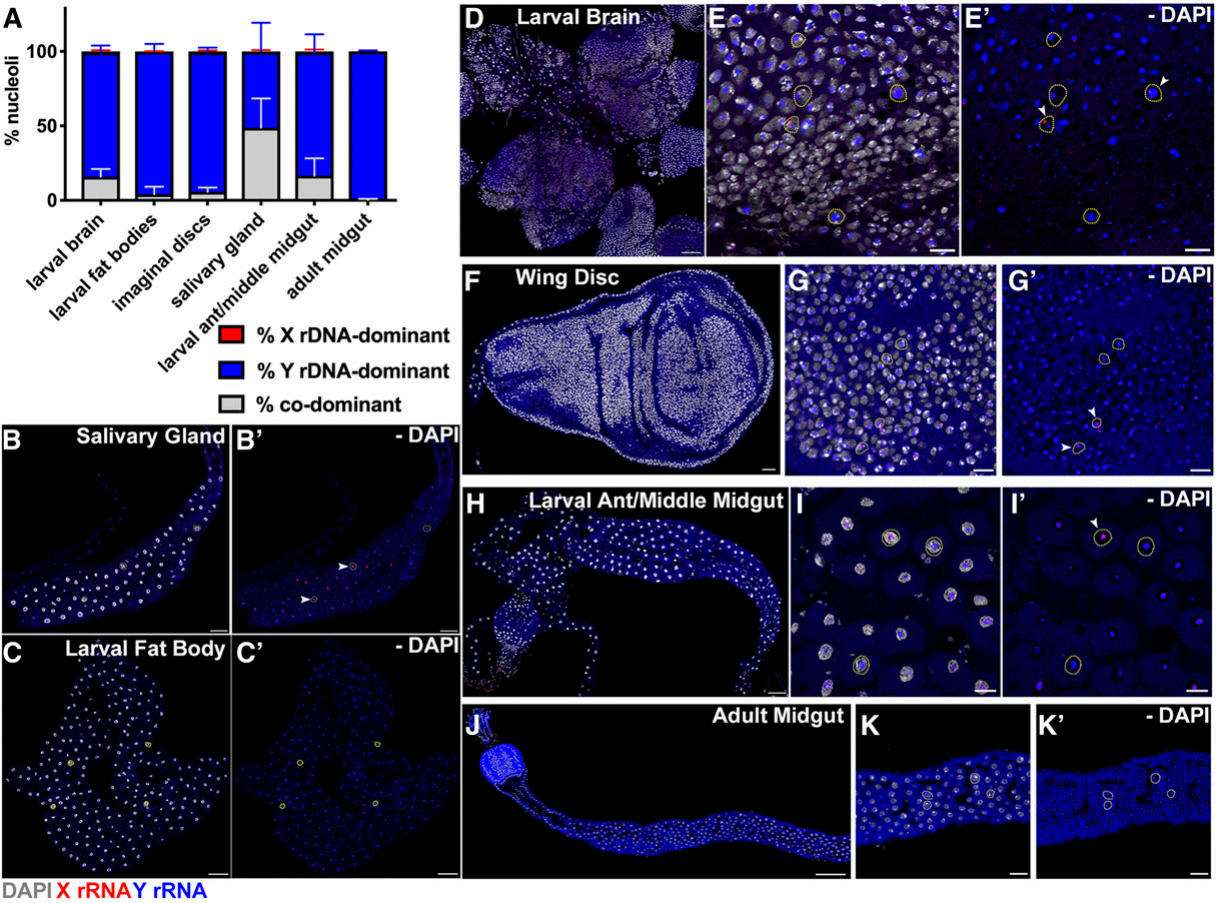
Y rDNA dominance is established during development in males. (A) Quantification of nucleolar dominance in larval and adult tissue(s) in males: larval brain (*n* = 1594 cells from six brains), larval fat bodies (*n* = 1575 cells from 17 fat bodies), imaginal discs (*n* = 1251 cells from five imaginal discs), salivary gland (*n* = 878 cells from 15 salivary glands), larval anterior/middle midgut (*n* = 81 cells from six guts), and adult anterior midgut (*n* = 922 cells from seven guts). Red = % *X* rDNA-dominant, blue = % *Y* rDNA-dominant, gray = % codominant. (B) Representative images of whole mount salivary gland, Bar, 100 μm. (B’) No DAPI. (C) Larval fat body, Bar, 100 μm. (C’) No DAPI. (D) Larval brain, Bar, 50 μm. (E) Zoomed image of larval brain, Bar, 10 μm. (E’) No DAPI. (F) Wing disc, Bar, 25 μm. (G) Zoomed image of wing disc, Bar, 8 μm. (G’) No DAPI. (H) Larval anterior midgut, Bar, 100 μm. (I) Zoomed image of larval anterior midgut, Bar, 25 μm. (I’) No DAPI. (J) Adult anterior midgut, Bar, 100 μm. (K) Zoomed image of adult anterior midgut, Bar, 25 μm. (K’) No DAPI. Red = *X* rRNA, blue = *Y* rRNA, white = DAPI. Yellow circles accent examples of SNP *in situ* signal in nuclei. Arrowheads indicate examples of codominant nuclei.

As development proceeds to the larval stage, we observed much higher rates of *Y* rDNA dominance in most tissues: larval brains (83.5 ± 4.6%) similar to what has been previously reported (Greil and Ahmad 2012), imaginal discs (93.6 ± 3.1%), larval fat bodies (95.8 ± 5.1%), and larval anterior midgut enterocytes (82.9 ± 12.1%) (Figure 2A, C–I). Salivary glands, which undergo a high degree of polyploidization, showed only a moderate degree of *Y* rDNA dominance (51 ± 19.4%) (Figure 2, A and B). *Y* rDNA dominance in the anterior midgut further increased in the adult (from 82.9% ± 12.1% in third instar larvae to 99.7 ± 0.7% in adult) (Figure 2, A, J, and K). These data suggest that nucleolar dominance in *D. melanogaster* males is gradually established over the course of development. This is similar to what was reported in *Brassica* (Chen and Pikaard 1997) and separately in *Arabidopsis* hybrids (Pontes *et al.* 2007; Earley *et al.* 2010), where seedling cotyledons exhibit codominance and nucleolar dominance is established in later stages of development depending on the tissue, suggesting conservation of this phenomenon whether it be within a species or in hybrids.

### Histone methyltransferase *Su(var)3-9* aids in the establishment of *Y* rDNA dominance in males across tissues

**Figure 3:**
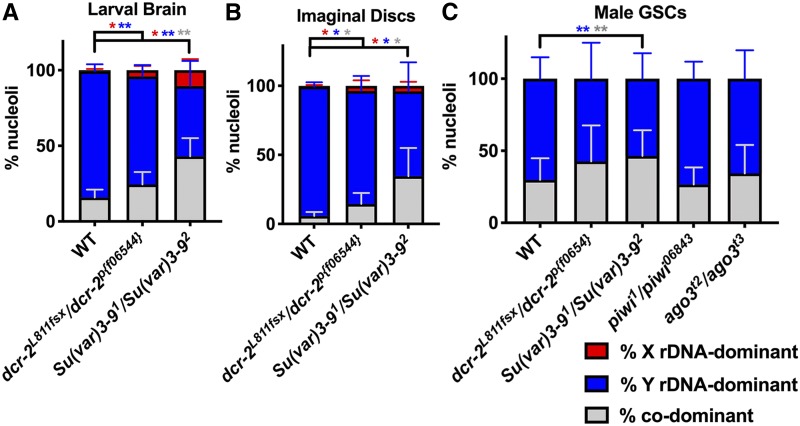
Heterochromatin formation aids in *Y* rDNA dominance in males. Quantification of male nucleolar dominance in (A) larval brains of wild type (*yw*) (*n* = 1594 cells from six brains), *dcr-2^L811fsx^/dcr dcr-2^p[f06544]^* mutants (*n* = 504 cells from six brains), and *Su(var)3-9^1^/Su(var)3-9^2^* mutants (*n* = 461 cells from six brains) (the same wild-type data from Figure 2 for comparison). (B) Quantification of nucleolar dominance in male imaginal discs of wild type (*n* = 1251 cells from five imaginal discs), *dcr-2^L811fsx^/dcr-2^p[f06544]^* mutants (*n* = 579 cells from nine imaginal discs), and *Su(var)3-9^1^/Su(var)3-9^2^* mutants (*n* = 432 cells from six imaginal discs) (the same wild-type data from Figure 2 for comparison). (C) Quantification of male nucleolar dominance in adult germline stem cells of wild type (*yw*) (*n* = 87 cells from 11 testes), *dcr-2^L811fsx^/dcr dcr-2^p[f06544]^* mutants (*n* = 122 cells from 15 testes), *Su(var)3-9^1^/Su(var)3-9^2^* mutants (*n* = 298 cells from 37 testes), *piwi*^*1*^*/piwi^06843^* mutants (*n* = 191 cells from 20 testes), and *ago3*^*t2*^*/ago3^t3^* mutants (*n* = 220 cells from 28 testes). Red = % *X* rDNA-dominant, blue = % *Y* rDNA-dominant, gray = % codominant nuclei. *P*-values calculated using Welch’s unpaired, unequal variances *t*-test using *n* = number of tissues. no star = not significant, * *P* < 0.05, ** *P* < 0.01. Colors of asterisks correspond to colors of bars for which *P*-values were calculated (*e.g.*, blue asterisk for *Y* rDNA-dominant *P*-values). WT, wild type.

siRNAs in *Arabidopsis* (Preuss *et al.* 2008) and long noncoding, promoter-associated RNAs in mammalian cell lines (Mayer *et al.* 2006) were shown to regulate rDNA silencing. These noncoding RNAs recruit factors that induce heterochromatinization of rDNA through DNA methylation (Lawrence *et al.* 2004; Preuss *et al.* 2008; Schmitz *et al.* 2010), histone methylation (Lawrence *et al.* 2004; Santoro and Grummt 2005; Pontvianne *et al.* 2012), and histone deacetylation (Santoro and Grummt 2005; Earley *et al.* 2006). The siRNA pathway and heterochromatin factors have also been shown to influence nucleolar morphology in *D. melanogaster* larval tissues, which may reflect disrupted rDNA expression (Peng and Karpen 2007). Based on these previous studies, we wondered whether the siRNA machinery and/or heterochromatin formation play a role in nucleolar dominance in *D. melanogaster*. To test this, we assessed nucleolar dominance in the mutants of *dicer-2* (*dcr-2*), an endonuclease critical for the siRNA pathway (Kim *et al.* 2006), and *Su(var)3-9*, a histone methyltransferase critical for depositing heterochromatin-associated histone methylation (Elgin and Reuter 2013). We found that *dcr-2^L811fsx^/dcr-2^p[f06544]^* mutants showed only a slight (although statistically significant) change in *Y* rDNA dominance in larval brains (71.4 ± 7.5% compared to control 83.5 ± 4.6%) and imaginal discs (81.8 ± 10.9% compared to control 93.6 ± 3.1%) (Figure 3, A and B, Figure S1, A, B, D, and E), and no change in adult GSCs (57.3 ± 25.0% compared to control 70.1 ± 14.9%) (Figure 3C), suggesting that the siRNA mechanism might not play an important role in nucleolar dominance, as opposed to what is reported in *Arabidopsis* (Pontes *et al.* 2006; Preuss *et al.* 2008). However, the potential redundancy between *dcr-2* and *dcr-1* could not be excluded as the *dcr-1* mutant was too sick to generate compound double mutants with *dcr-2* (*dcr-1/+*; *dcr-2/+*).

*Su(var)3-9^1^/Su(var)3-9^2^* mutants showed a marked decrease in *Y* rDNA dominance in larval brain (46.5 ± 16.5%, compared to control 83.5 ± 4.6%), imaginal discs (61.4 ± 21.0%, compared to control 93.6 ± 3.1%), and adult GSCs (53.5 ± 17.7%, compared to control 70.1 ± 14.9%) (Figure 3, A–C, Figure S1, A, C, D, and F), consistent with the previous finding that *Su(var)3-9* is involved in silencing of *X* rDNA in male neuroblasts (Greil and Ahmad 2012). *dicer-2* and *Su(var)3-9* mutants had minimal effects on nucleolar dominance in polyploid tissues (salivary glands, larval fat bodies, larval anterior midgut, and adult anterior midgut enterocytes) (Figure S2). These results suggest that the siRNA pathway does not play a major role in silencing of *X* rDNA for establishment of nucleolar dominance, whereas heterochromatin formation mediated by *Su(var)3-9* is important for nucleolar dominance of diploid tissues in *D. melanogaster* males.

We further examined Piwi-interacting RNA (piRNA) pathway components in the germline, as the piRNA pathway is an important silencing mechanism in the germline (Yamashiro and Siomi 2018). However, neither *piwi* (*P*-element-induced wimpy testes) or *ago3* (*argonaute 3*) mutants compromised nucleolar dominance in male GSCs (Figure 3C). Piwi and Ago3 are involved in the nuclear and cytoplasmic arms of the piRNA pathway, respectively (Yamashiro and Siomi 2018). The GSCs of *piwi*^*1*^*/piwi^06843^* males showed no change in *Y* rDNA dominance (73.4 ± 11.8% compared to wild-type control 70.1 ± 14.9%) (Figure 3C). Likewise, *ago3*^*t2*^/*ago3*^*t3*^ mutants did not change rates of *Y* rDNA dominance (65.7 ± 19.7% compared to wild-type control 70.1 ± 14.9%) (Figure 3C). These results suggest that the piRNA pathway does not play a major role in silencing of *X* rDNA in nucleolar dominance in male GSCs.

### Codominance is commonly observed in *X/X* female tissues

**Figure 4:**
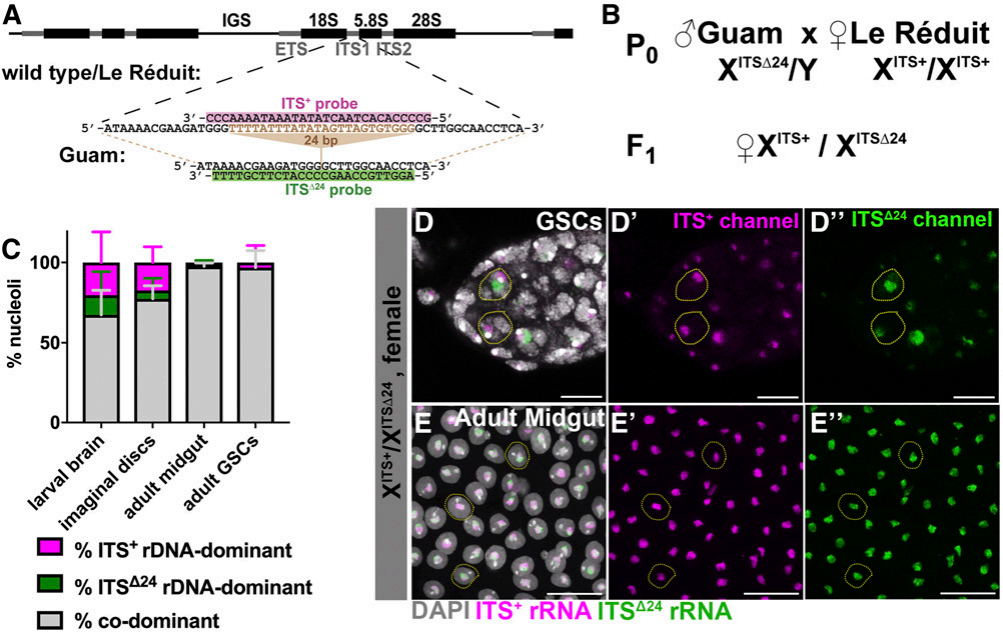
*X/X* females express both rDNA loci throughout development. (A) Oligonucleotide probe design to differentially visualize two distinct *X* chromosome rDNA internal transcribed spacer (ITS) transcripts, utilizing a 24-bp deletion in the rDNA ITS between wild-type/Le Réduit and Guam *D. melanogaster* strains. (B) The cross to generate female F1 with one *X* chromosome with wild-type ITS (ITS^+^) and the other *X* chromosome with the ITS with 24 bp deletion (ITS^Δ24^). (C) Quantification of nucleolar dominance between two *X* rDNA in female larval and adult tissue(s): larval brain (*n* = 2616 cells from nine brains), imaginal discs (*n* = 2575 cells from nine imaginal discs), adult anterior midgut (*n* = 904 cells from nine guts), and adult GSCs (*n* = 150 cells from 57 germarium). Magenta = % ITS^+^ rDNA-dominant, green = % ITS^Δ24^ rDNA-dominant, gray = % codominant nuclei. Representative images of (D–D’’) GSCs, Bar, 8 μm, and (E–E’’) adult anterior midgut enterocytes, Bar, 25 μm. Magenta = ITS^+^ rRNA, green = ITS^Δ24^ rRNA, white = DAPI.

It was previously shown that nucleolar dominance does not occur in female larval neuroblasts (Durica and Krider 1977; Greil and Ahmad 2012). We sought to determine the state of nucleolar dominance in females (between two *X* rDNA loci) across tissues and developmental stages. Doing so requires two distinct *X* rDNA loci with detectable differences, similar to SNP *in situ* hybridization described above for *X vs. Y* rDNA. Our initial searches for SNPs between *X* rDNA loci from multiple laboratory strains revealed no SNPs (see *Materials and Methods* and the Reagents Table). However, sequencing of *X* rDNA from geographically separated *D. melanogaster* strains led us to the identification of a 24-bp deletion in the internal transcribed spacer (ITS1) of the *X* rDNA in a strain originating from Guam, compared to most other strains sequenced (*i.e.*, *yw*, Oregon-R, Canton-S, Beijing, Pohnpei, Samoa, Port Moresby, Le Réduit) (see *Materials and Methods*; Figure 4A). We designed oligonucleotide probes to distinguish the rRNA from the Guam strain (ITS^Δ24^) *vs.* other strains (ITS^+^) (see *Materials and Methods*; Figure 4A). The Guam strain exhibited signals only from ITS^Δ24^ rDNA (Figure S3). Among other strains that have the ITS^+^ variant, the Le Réduit strain had the least background signal with the ITS^Δ24^ probe (Figure S3), whereas females from other strains revealed weak ITS^Δ24^ signal in addition to predominant ITS^+^ signal (data not shown), possibly because these strains may contain a small fraction of rDNA copies with the ITS^Δ24^ variant. Based on these results, we decided to utilize the Guam and Le Réduit strains to determine the state of nucleolar dominance between two *X* rDNA loci in females.

We crossed Guam strain males with Le Réduit strain females and tissues from the resulting F1 females were assessed for the state of nucleolar dominance by RNA *in situ* using the ITS^Δ24^ and ITS^+^ probes (Figure 4B). We found that *X/X* female cells predominantly show expression from both rDNA loci (*i.e.*, codominant) in larval brains (67.1 ± 15.6% codominant) (Figure 4C). We found that *X/X* female larval imaginal discs also exhibit primarily codominance (77.3 ± 8.1%) (Figure 4C). Adult tissues revealed even higher rates of codominance compared to larval tissues: anterior midgut enterocytes (97.6 ± 2.1%) and GSCs (96.8 ± 10.7%) (Figure 4, C–E). It should be noted that we did not assess nucleolar dominance in female embryos because the Guam strain *Y* rDNA shares the same ITS sequence as Le Réduit *X* rDNA (ITS^+^), making the accurate sexing of embryos impossible. However, female embryos in the experiments described in Figure 1, I–L mostly exhibited two nucleoli per nucleus, suggesting that female embryos also exhibit codominance.

The codominant state of *X* rDNA loci in the progeny of Guam and Le Réduit parents did not change even when parental origin of ITS^Δ24^ *vs.* ITS^+^ rDNA loci was switched (*i.e.*, Guam females crossed to Le Réduit males) (Figure S4B). It should be noted that in this direction of the cross, females exhibited hybrid dysgenesis, leading to a high frequency of degenerated ovaries (Figure S4A). Despite high rates of hybrid dysgenesis, all cells scored exhibited codominance (Figure S4B). These results establish that females exhibit codominance in a broad range of tissues and developmental stages, extending the previous findings in female neuroblasts (Durica and Krider 1977; Greil and Ahmad 2012).

### *Y* rDNA dominates in female cells

The above results reveal a striking difference in the state of nucleolar dominance between males and females: *Y* rDNA dominates over *X* rDNA in males, whereas two *X* chromosomes are codominant in females. What accounts for this striking difference in the state of nucleolar dominance between males and females? A previous study showed that nucleolar dominance in *D. melanogaster* is not likely due to imprinting during the parents’ gametogenesis, as reversing inheritance of *X vs. Y* chromosomes (*i.e.*, *X* from father, *Y* from mother) did not influence the state of *Y* rDNA dominance (Greil and Ahmad 2012). Others have speculated that distinct sequence differences between the loci, in this case the *X* rDNA *vs. Y* rDNA loci, allow selective expression/silencing of particular rDNA loci (Kidd and Glover 1981; Macleod and Bird 1982; Labhart and Reeder 1984; Grimaldi *et al.* 1990; Heix and Grummt 1995; Neves *et al.* 1995; Houchins *et al.* 1997; Caudy and Pikaard 2002; Felle *et al.* 2010). Yet another possibility is that chromosomal context, or location within a particular chromosome (Chandrasekhara *et al.* 2016; Mohannath *et al.* 2016), may determine whether or not a particular rDNA locus may be expressed/silenced. In addition, cellular sex might determine whether or not nucleolar dominance occurs.

**Figure 5:**
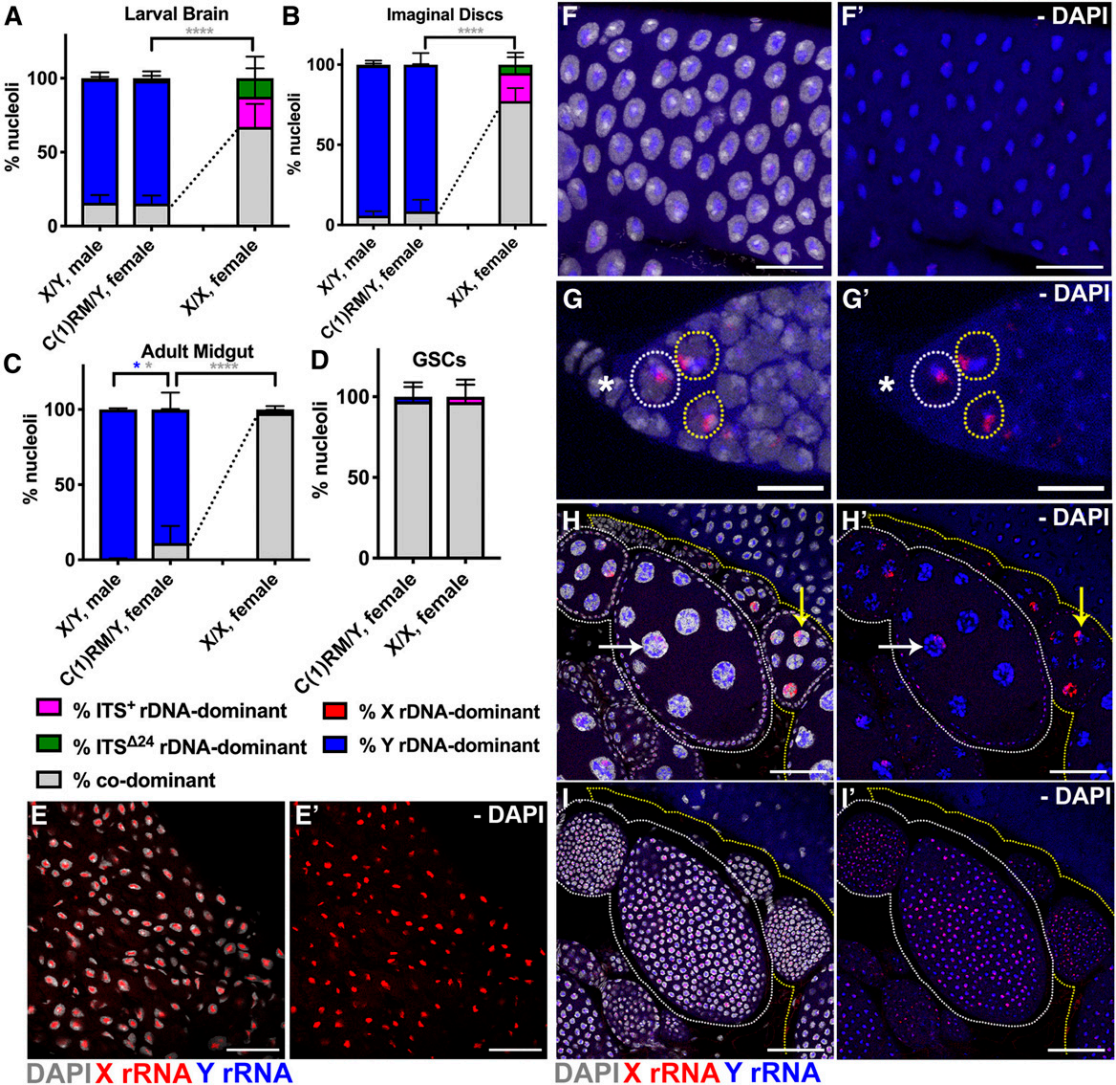
The *Y* rDNA can dominate over *X* rDNA in female cells. (A–D) Quantification of nucleolar dominance in C(1)RM/Y females compared to males (data from Figure 2 for comparison) and typical *X/X* females (data from Figure 4 for comparison) in larval brain [(A), *n* = 914 cells from nine brains], imaginal discs [(B), *n* = 1068 cells from 12 imaginal discs], adult anterior midgut enterocytes [(C), *n* = 870 cells from seven guts], and female GSCs [(D), *n* = 54 cells from 21 germarium]. Dotted lines denote differences in rates of codominance between C(1)RM/Y and typical *X/X* females. *P*-values calculated using Welch’s unpaired, unequal variances *t*-test using number of tissues scored. *P*-values between C(1)RM/Y and *X/X* females were only calculated for % codominant. no star = not significant, * *P* < 0.05, **** *P* < 0.0001. Colors of asterisks correspond to colors of bars for which *P*-values were calculated (*e.g.*, blue asterisk for *Y* rDNA-dominant *P*-values). (E) Representative control image of C(1)RM female adult midgut enterocytes, Bar, 50 μm. (E’) No DAPI. (F) Representative image of C(1)RM/Y female adult anterior midgut enterocytes, Bar, 25 μm, (F’) No DAPI. (G) C(1)RM/Y female GSCs (white circle) and cystoblasts (yellow circles), * indicates terminal filament, Bar, 10 μm. (G’) No DAPI. (H) Two C(1)RM/Y ovarioles (separately circled in white or yellow), Bar, 50 μm. (H’) No DAPI. Arrows indicate nurse cells with low *X* rDNA expression (white) and high *X* rDNA expression (yellow). (I) Follicle cells from C(1)RM/Y ovarioles corresponding to (H) (different *Z*-depth), Bar, 50 μm. (I’) No DAPI. Red = *X* rRNA, blue = *Y* rRNA, white = DAPI.

Because parental imprinting unlikely contributes to the regulation of nucleolar dominance (Greil and Ahmad 2012), we sought to test the possibility that *X* and/or *Y* rDNA contain specific elements that determine the state of nucleolar dominance. To this end, we examined the state of nucleolar dominance in females that carry a *Y* chromosome. C(1)RM is a compound *X* chromosome (two *X* chromosomes are fused and it contains *X* rDNA) and C(1)RM/Y flies develop as females (Bridges 1916; Bridges 1921). The rDNA on C(1)RM was found to share all SNPs with the *yw X* rDNA (see *Materials and Methods* and the Reagent Table) and the *Y* rDNA SNP *in situ* probes did not cross-hybridize (Figure 5E). Utilizing these SNPs, we determined the state of nucleolar dominance between C(1)RM rDNA and *Y* rDNA in female tissues (*e.g.*, diploid larval tissues, the adult anterior midgut, and adult ovary). Surprisingly, C(1)RM/Y females exhibited a strikingly high frequency of *Y* rDNA dominance in many cell types (Figure 5, A–C and F). This is in stark contrast to *X/X* females, where two *X* chromosomes exhibit codominance across tissues (Figure 5, A–D). These results indicate that nucleolar dominance is determined by the presence of a *Y* chromosome (*e.g.*, sequence information within *Y* rDNA or other elements on the *Y* chromosome), and disfavors the possibility that cellular sex determines whether or not nucleolar dominance occurs.

Interestingly, adult GSCs and cystoblasts showed a high degree of codominance in C(1)RM/Y females (Figure 5, D and G) (GSCs: 97.2 ± 8.9% codominance), whereas some nurse cells showed *Y* rDNA dominance (Figure 5H). The somatic follicle cells of the egg chambers showed mostly codominance (Figure 5I). These data together suggest that, whereas the *Y* rDNA dominates irrespective of cellular sex, it is not the sole factor to determine nucleolar dominance, and that cell-type-specific information may modulate the state of nucleolar dominance.

### Element(s) within the *Y* chromosome contribute to nucleolar dominance

**Figure 6:**
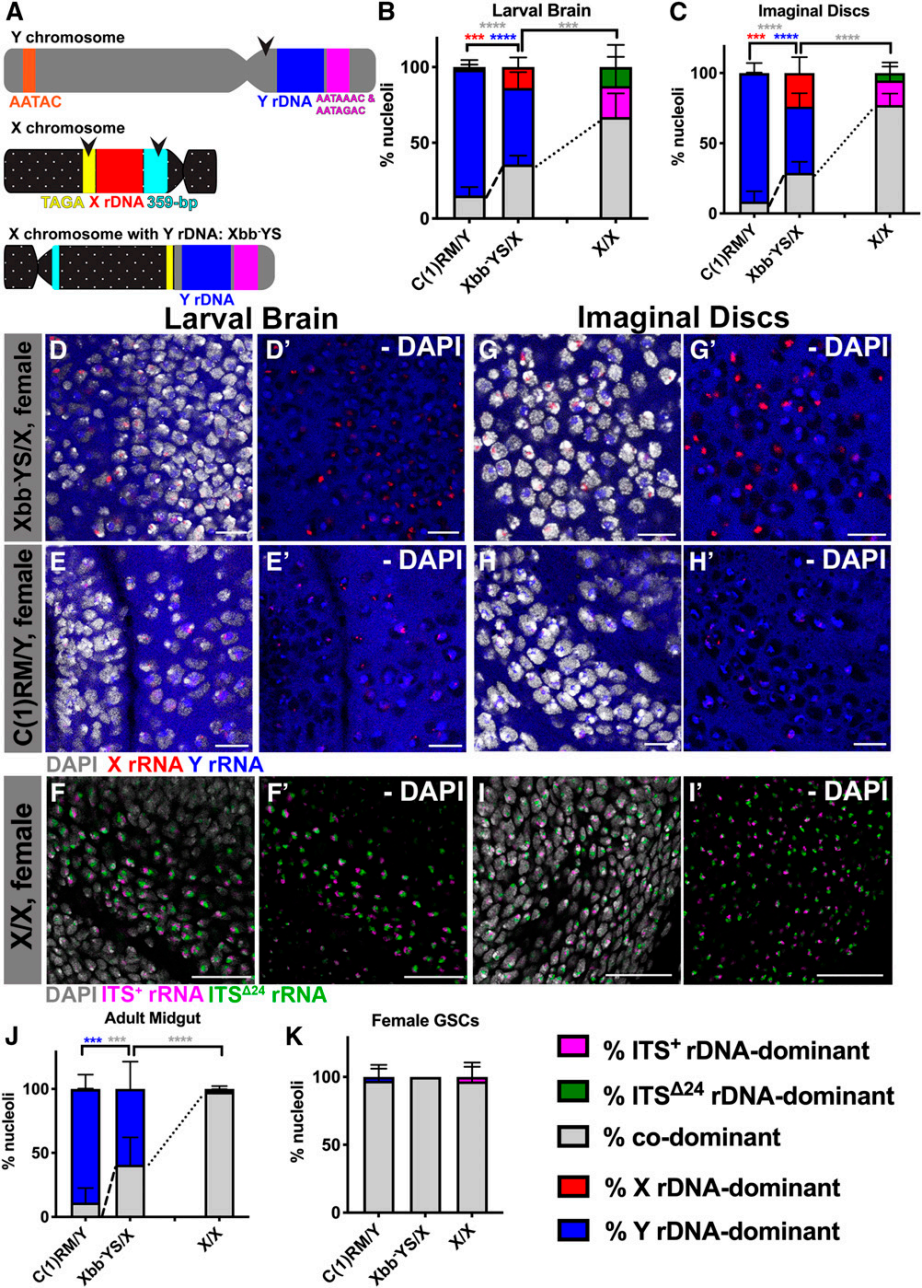
*Y* rDNA likely has information to dictate nucleolar dominance. (A) Structure of a wild-type *Y* chromosome, a wild-type *X* chromosome, and the Xbb^−^YS chromosome based on Figure S5. Constriction represents centromere location. Black arrows mark location of chromosome break. (B and C) Quantification of nucleolar dominance in Xbb^−^YS/X females compared to C(1)RM/Y females (data from Figure 5 for comparison) and *X/X* females (data from Figure 4 for comparison) in larval brain [(B), *n* = 1400 cells from eight brains] and imaginal discs [(C), *n* = 1362 cells from nine imaginal discs]. Red = % *X* rDNA-dominant, blue = % *Y* rDNA-dominant, gray = % codominant, magenta = % ITS^+^ rDNA-dominant, green = % ITS^Δ24^ rDNA-dominant nuclei. *P*-values calculated using Welch’s unpaired, unequal variances *t*-test using *n* = number of tissues. *P*-values between Xbb^−^YS/X and *X/X* females were only calculated for % codominance. Dotted lines denote differences in rates of codominance between Xbb^−^YS/X and *X/X* females. Dashed lines denote differences in rates of codominance between Xbb^−^YS/X females and C(1)RM/Y females. *** *P* < 0.001, **** *P* < 0.0001. Colors of asterisks correspond to colors of bars for which *P*-values were calculated (*e.g.*, blue asterisk for *Y* rDNA-dominant *P*-values). Representative images of larval brain from (D) Xbb^−^YS/X females, Bar, 10 μm; (D’) no DAPI; (E) C(1)RM/Y females, Bar, 10 μm; (E’) no DAPI; (F) *X/X* females, Bar, 25 μm; and (F’) no DAPI. Representative images of imaginal discs from (G) Xbb^−^YS/X females, Bar, 8 μm; (G’) no DAPI; (H) C(1)RM/Y females, Bar, 10 μm; (H’) no DAPI; (I) X/X females, Bar, 25 μm; and (I’) no DAPI. Red = *X* rRNA, blue = *Y* rRNA, white = DAPI, magenta = ITS^+^ rDNA transcript, green = ITS^Δ24^ rDNA transcript. (J) Quantification of nucleolar dominance in Xbb^−^YS/X females compared to both C(1)RM/Y females (data from Figure 5 for comparison) and *X/X* females (data from Figure 4 for comparison) in adult anterior midgut enterocytes (*n* = 1213 cells from 13 guts), and (K) female GSCs (*n* = 122 cells from 51 germarium). Red = % *X* rDNA-dominant, blue = % *Y* rDNA-dominant, gray = % codominant, magenta = % ITS^+^ rDNA-dominant, green = % ITS^Δ24^ rDNA-dominant nuclei. *P*-values calculated using Welch’s unpaired, unequal variances *t*-test. *P*-values between Xbb^−^YS/X and *X/X* females were only calculated for % codominance. Dotted lines denote differences in rates of codominance between Xbb^−^YS/X and *X/X* females. Dashed lines denote differences in rates of codominance between Xbb^−^YS/X females and C(1)RM/Y females. no star = not significant, *** *P* < 0.001, **** *P* < 0.0001.

The above data that the *Y* rDNA dominates over the *X* rDNA irrespective of cellular sex in most cell types indicate that the *Y* chromosome may contain element(s) that regulate nucleolar dominance. Such information may be embedded in the *Y* rDNA locus itself, such as variable sequences in the coding and/or spacer sequences (Tautz *et al.* 1987; Tautz *et al.* 1988; Schlötterer *et al.* 1994; Caudy and Pikaard 2002). Additionally, the entire chromosome in which the rDNA is located may dictate the state of silencing/activation (Chandrasekhara *et al.* 2016; Mohannath *et al.* 2016). To address whether the *Y* rDNA contains information that influences its dominance, we utilized an *X* chromosome that contains *Y* rDNA due to chromosomal rearrangements. This chromosome, In(1)sc^4L^sc^8R^ + Tp(1; YS)bb^AM7^(referred to as Xbb^−^YS), lacks *X* rDNA (Xbb^−^) but instead contains *Y* rDNA together with other element(s) of the *Y* chromosome short arm (YS) (Rasooly and Robbins 1991) (Figure 6A, Figure S5). We first sequenced the rDNA from the Xbb^−^YS chromosome and found that its rDNA exhibited three SNPs compared to *yw X* rDNA (see *Materials and Methods* and the Reagent Table). Using these three sets of SNP *in situ* probes, we found that Xbb^−^YS/X females exhibit intermediate patterns of nucleolar dominance: in larval brain, imaginal discs, and adult anterior midgut enterocytes, Xbb^−^YS rDNA mostly dominates over *X* rDNA, as opposed to codominance in typical *X/X* females (Figure 6, B–J). However, the degree of Xbb^−^YS rDNA dominance was lower than *Y* rDNA dominance in *X/Y* males (Figure 6, B–J) and in C(1)RM/Y females (Figure 6, B–J). GSCs from Xbb^−^YS/X females exhibited high rates of codominance, similar to *X/X* females (Figure 6K) as well as C(1)RM/Y females (compare to Figure 5D). These results suggest that *Y* rDNA and/or its proximal region within YS carries critical information that allows for establishment of nucleolar dominance, where *Y* rDNA is transcribed and *X* rDNA is silenced. This is similar to what has been seen in *Arabidopsis*, where nucleolar dominance is “allelic” and possibly influenced by rDNA sequence differences (Rabanal *et al.* 2017). Additionally, the observation that the degree of *Y* rDNA dominance in Xbb^−^YS/X females is much less than that in *X/Y* males or C(1)RM/Y females indicates that the chromosomal context (Chandrasekhara *et al.* 2016) (*e.g.*, being embedded in the entire *Y* chromosome) and/or with other factor(s) on the long arm of the *Y* chromosome (YL) also play a role in the determination of *Y* rDNA dominance (see *Discussion*).

## Discussion

In this study, we conducted a thorough characterization of nucleolar dominance in the context of nonhybrid *D. melanogaster*. Our study extends the previous discovery in *D. melanogaster* male larval neuroblasts that nucleolar dominance occurs within a species (Greil and Ahmad 2012; Zhou *et al.* 2012) to a broader range of tissues and developmental stages. Our study shows that nucleolar dominance is a developmentally regulated process, being established gradually during the course of development. This is reminiscent of what was seen in *Brassi**ca* and *Arabidopsis* (Chen and Pikaard 1997; Pontes *et al.* 2007; Earley *et al.* 2010), and supports the notion that nucleolar dominance is not limited to interspecies hybrids.

Earlier studies (Lawrence *et al.* 2004; Santoro and Grummt 2005; Earley *et al.* 2006; Earley *et al.* 2010; Greil and Ahmad 2012; Pontvianne *et al.* 2012), confirmed here, revealed heterochromatin formation as a critical aspect of nucleolar dominance. The mechanism through which heterochromatin factors are assembled to the silenced rDNA locus appear to vary across species: siRNAs direct silencing marks in *Arabidopsis* (Preuss *et al.* 2008), long promoter associated RNAs silence rDNA in mammalian cell lines (Santoro *et al.* 2010), and rDNA silencing in *Drosophila* does not appear to involve siRNAs or piRNAs. Although these studies reveal the need of heterochromatinization to silence rDNA loci that were chosen to be silenced, they do not provide the mechanism of “choice” that dictates which particular rDNA loci are to be silenced or activated.

In this regard, elements within rDNA have been suggested to influence the “choice” of nucleolar dominance, particularly the intergenic spacer sequence (IGS), which contains rDNA promoters (Coen and Dover 1982) and enhancer function (Labhart and Reeder 1984). Different species’ IGS was shown to compete for rDNA transcription factors where longer spacers outcompete short spacers in *Xenopus laevis* oocytes (Reeder *et al.* 1983; Labhart and Reeder 1984). This difference in transcription factor binding based on spacer length was speculated to influence nucleolar dominance in interspecies *Xenopus* hybrids since larger *X. laevis* spacers are able to dominate over the short *X. borealis* spacers when injected into either species’ oocyte (Reeder and Roan 1984). Because the IGS sequences are known to be highly diverged compared to the coding region of rDNA (Tautz *et al.* 1987; Tautz *et al.* 1988), it is tempting to speculate that differences in IGS sequences between *X* and *Y* rDNA loci can differentiate two loci to instruct nucleolar dominance. However, differences in IGS sequences in *Brassi**ca** rapa* and *B. oleracea* did not dictate nucleolar dominance in their hybrid, *B. napus* (Frieman *et al.* 1999), suggesting that there may be a considerable variation in the mechanisms that instruct nucleolar dominance across species. Modifying the large and highly repetitive IGS (∼200–250 copies/chromosome) to test these possibilities remains a technical challenge.

Elements within heterochromatin adjacent to the rDNA locus have been shown to influence nucleolar dominance in interspecies hybrids between *D. melanogaster* and *D. simulans*. Durica and Krider found that the YL with rDNA was not able to induce nucleolar dominance over *D. simulans* rDNA (Durica and Krider 1978). The YS with rDNA was able to induce nucleolar dominance, but to a lesser extent than a complete *Y* chromosome (Durica and Krider 1978). This suggests that YS has a stronger influence on nucleolar dominance than YL, but YL may contain some element(s) that aids in complete establishment of nucleolar dominance in interspecies hybrids. These findings in *Drosophila* hybrids are similar to what we report in nonhybrid female *D. melanogaster*, where YS with rDNA exhibits a strong influence on its dominance over the *X* rDNA, albeit incomplete (Figure 6), suggesting that the underlying mechanism of nucleolar dominance in nonhybrids *vs.* interspecies hybrids may be similar in *Drosophila*, as suggested by studies of *Arabidopsis*. In experiments described in this study, where the entire or portion of the *Y* chromosome was introduced into the context of females [C(1)RM/Y and Xbb^−^YS/X], the *Y* rDNA exhibited a high degree of dominance even in female cells, suggesting that the *Y* chromosome harbors certain information that dictates *Y* rDNA transcription and/or *X* rDNA silencing. It remains elusive if the element(s) of the *Y* chromosome are selectively activating the *Y* rDNA (acts in *cis*) and/or selectively suppressing *X* rDNA transcription (acts in *trans*). However, considering the involvement of Su(var)3-9 in nucleolar dominance (Figure 3), it is likely that the *Y* chromosome contains the information to dictate the silencing of *X* rDNA. Whether the *Y* chromosome also has information to simultaneously maintain its own transcription remains unknown.

Most importantly, why a locus-wide mechanism, *i.e.*, nucleolar dominance, has evolved to regulate rDNA expression is a fundamental question. Our previous study revealed preferential copy number loss from the *Y* rDNA in male GSCs during aging (Lu *et al.* 2018), potentially as a result of intrachromatid recombination occurring during active transcription (Takeuchi *et al.* 2003). Once *Y* rDNA copy number is reduced, *X* rDNA became active, shifting toward codominance (Lu *et al.* 2018). These observations may point to a possibility that nucleolar dominance may protect the *X* rDNA locus from degeneration, and may serve as a mechanism to monitor and regulate rDNA copy number.

In summary, our work expands on previous studies in *Arabidopsis* and *Drosophila* and supports the notion that nucleolar dominance is not constrained to interspecies hybrids, and represents a mechanism of rRNA regulation within a species. Our study suggests that the *Y* rDNA and/or the YS may have elements that dictate *Y* rDNA’s dominance (either by *X* rDNA silencing and/or *Y* rDNA activation) in *D. melanogaster*. The precise identity of the element(s) of the *Y* rDNA/YS, and how they mediate nucleolar dominance (whether via preferential transcription of *Y* rDNA and/or silencing of *X* rDNA) await future investigation. Our study lays the foundation to identify element(s) within and outside of the *Y* rDNA that regulate nucleolar dominance and to understand the underlying mechanisms needed to achieve nucleolar dominance.
